# Mitral Valve Infective Endocarditis Complicated With Coronary Artery Embolization

**DOI:** 10.7759/cureus.21459

**Published:** 2022-01-20

**Authors:** Cristina Carvalho Gouveia, Inês Pimenta, Marco Fernandes, Beatriz Chambino, Hugo Côrte-Real

**Affiliations:** 1 Internal Medicine, Hospital São Francisco Xavier, Lisbon, PRT; 2 Intensive Care, Hospital Garcia de Orta, Almada, PRT; 3 Intensive Care, Hospital Santa Maria, Lisbon, PRT

**Keywords:** coronary revascularization, mitral valve replacement, coronary artery septic embolization, systemic embolization, infective endocarditis

## Abstract

Infective endocarditis is a multisystem and potentially fatal disease. Systemic embolization is a relatively common complication, the spleen and central nervous system being the most frequent sites for septic emboli formation. Coronary artery septic embolization is extremely uncommon and its management remains controversial.

We present the case of a 50-year-old male diagnosed with mitral valve infective endocarditis complicated with spleen and central nervous system embolization, who developed acute myocardial infarction two weeks after disease onset. The patient was successfully treated with combined mitral valve replacement and coronary artery bypass grafting.

## Introduction

Infective endocarditis (IE) is a multisystem disease that results from an infection of a heart valve (native or prosthetic), endocardial surface, or indwelling cardiac devices [1-3]. Systemic embolization, a life-threatening complication of IE related to the migration of cardiac vegetations, occurs in 20-50% of patients [4-6]. The most common sites for embolization are the brain and spleen in left-sided IE and the pulmonary artery in right-sided IE [4]. Coronary artery septic embolization is a rare and potentially fatal complication of IE [5,7]. It occurs in 0.31-0.51% of patients with IE and represents 1.5-3.5% of all embolic events [5]. Because of its rarity, the most appropriate treatment remains to be defined [7]. 

## Case presentation

**Figure 1:**
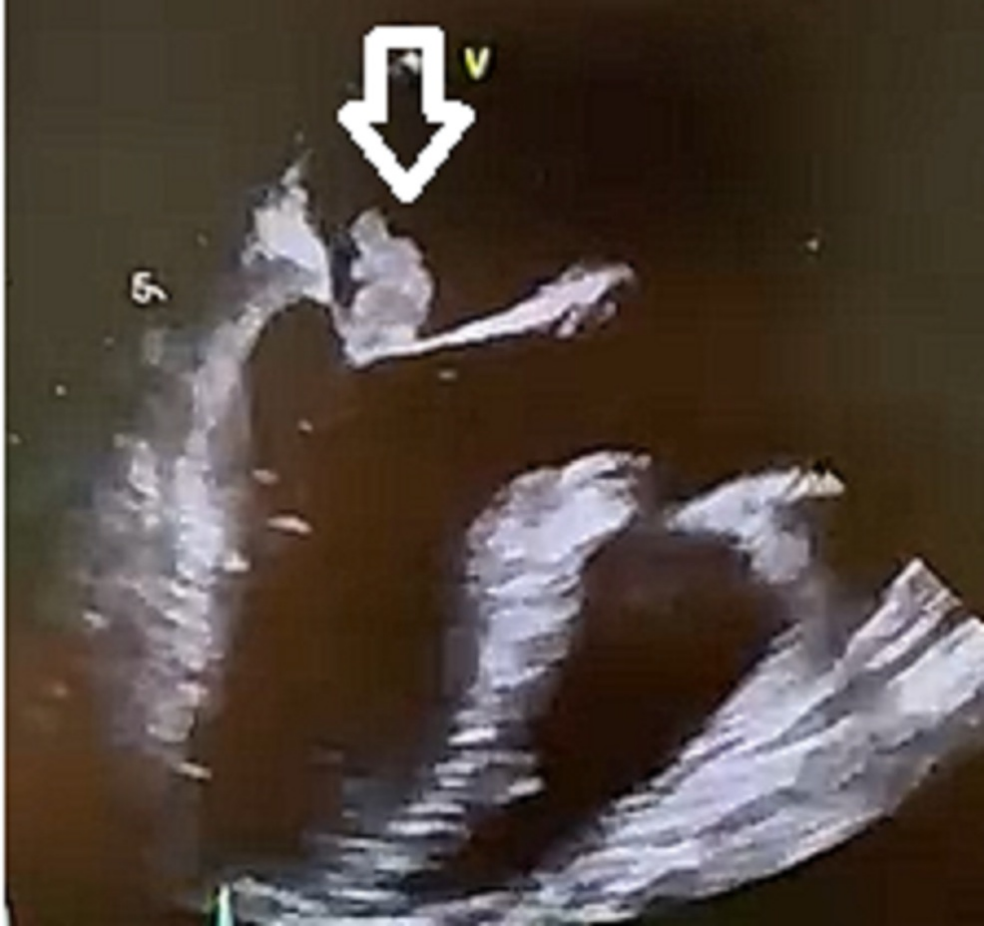
Echocardiogram Presence of mitral valve vegetation (arrow)

A 50-year-old male with a history of well-controlled arterial hypertension was admitted to an intensive care unit with symptoms of fever, myalgia, anorexia, asthenia, and disorientation. He was stung by a sea urchin in the right hand three days before symptom onset. The patient reported no respiratory, gastrointestinal, or urinary symptoms and denied recent travels or contact with other animals. On physical examination, he was hemodynamically stable, febrile (40º Celsius), disoriented, had an infected wound in the right hand, and had a systolic murmur in the mitral area. Besides the disorientation, the neurological examination was normal. Laboratory results revealed elevated leukocyte count (14.6 x 10^9/L) and C-reactive protein (19.2mg/dL). The transthoracic echocardiography (TTE) revealed vegetation in the anterior leaflet of the mitral valve, associated with severe valvular regurgitation and normal left ventricular systolic function (60%). The transesophageal echocardiography (TEE) was consistent with the TTE, showing an 18x10mm vegetation in the mitral valve (Figure 1).

The electrocardiogram (ECG) and coronary angiography were normal. The cranioencephalic computed tomography (CT) revealed tenuous cortico-subcortical hypodensities in the right parietal and left frontal areas. In order to investigate other potential foci of septic embolization, a thoraco-abdominopelvic CT was done, which revealed two areas of splenic infarction. 

**Figure 2:**
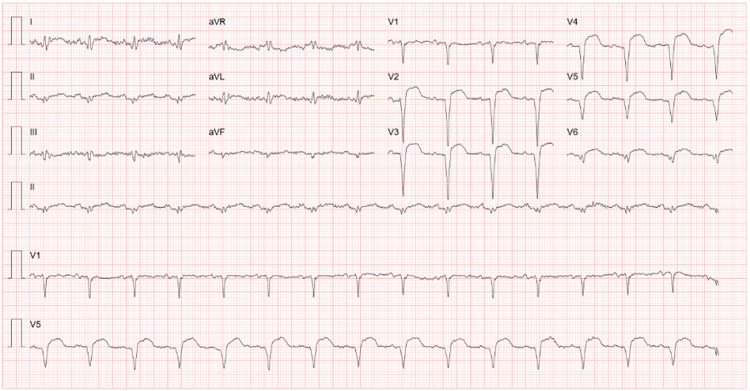
ECG ST elevation in leads V2 to V6 suggests an anterior wall myocardial infarction

The patient was diagnosed with infective endocarditis of the native mitral valve and severe mitral regurgitation, complicated with central nervous system and splenic embolization. He was started on ceftriaxone, azithromycin, and gentamicin after blood cultures were drawn. Twelve days after hospital admission, a routine ECG revealed signs of anterior wall myocardial infarction (Figure 2).

The patient had no symptoms suggestive of coronary ischemia such as chest pain, dyspnea, nausea, or diaphoresis. The TTE revealed acute depression of left ventricular systolic function (44%) and akinesia of the apex and mid-anteroseptal part of the interventricular septum. The coronary angiography showed a subocclusive lesion in the middle segment of the left anterior descending coronary artery (LAD). The definite diagnosis of mitral valve IE with central nervous system, splenic and coronary artery septic embolization was attained.

The patient was submitted to mechanical mitral valve replacement and coronary artery bypass grafting (left internal mammary artery to LAD), in order to address both situations. After the surgery, he was started on warfarin. The surgery and postoperative period were complicated by cardiogenic shock, which was managed with norepinephrine and dobutamine for five days.

All the blood cultures drawn before and during the treatment were negative, as were the culture and biopsy of the mitral valve. Because the patient reported being bitten by a sea urchin before disease onset,* Staphylococcus* spp. and *Streptococcus* spp. were considered the most probable etiological agents. The patient showed clinical and laboratory improvement and a decision was made to switch the antibiotic treatment to ceftriaxone and ampicillin. After completing four weeks of combined treatment with ceftriaxone and ampicillin, he was discharged home and was instructed to take linezolid for an additional two weeks, in order to complete six weeks of antimicrobial therapy.

## Discussion

Infective endocarditis has an incidence of 3-10 per 100 000 people [3]. *Staphylococcus aureus* is the most prevalent etiological agent (26.6-40%), followed by viridans group streptococci and* Enterococcus* spp [1,8]. Risk factors for IE include intravenous drug use, presence of prosthetic heart valves, indwelling catheters and cardiac devices, rheumatic heart disease, diabetes, immunosuppression, and congenital heart disease [2,3]. Diagnosis is based on clinical, microbiological, and imaging findings, which are included in the modified Duke clinical diagnostic criteria [2,3].

The clinical presentation of IE is varied and non-specific, fever and the presence of a cardiac murmur being the most common findings [3]. Other presentations include rigors, malaise, fatigue, weight loss, splenomegaly, cutaneous manifestations (petechiae, hemorrhages), and signs of complications, such as heart failure, embolization, sepsis, and metastatic infections [3,8].

Echocardiography is essential for the diagnosis of IE and the detection of complications [3,8]. TTE has a sensitivity of 70% in detecting vegetations in native valves, while the sensitivity and specificity of TEE are above 90% [1,8]. Echocardiographic manifestations of IE include valvular vegetation, abscess, fistula, leaflet perforation, valvular regurgitation, and prosthetic valve dehiscence [2]. Laboratory tests usually reveal elevated inflammatory and infective markers [3].

Empirical antibiotic regimens for native valve endocarditis are covered in guidelines [4]. Treatment should include bactericidal antibiotics for a prolonged period and be adjusted according to the pathogen, resistance patterns, severity of infection, type (native or prosthetic), and location of the affected valve [3,4,8].

Up to 50% of patients require surgery, the main indications being acute heart failure, uncontrolled infection, and prevention of septic embolization [1]. The in-hospital mortality for IE is up to 30% and the risk of recurrence is 2-6% [1].

Septic embolization occurs in up to 50% of cases, 25% of which are at presentation [1,2]. Risk factors for embolization are size (>10mm) and mobility of vegetations, location on the mitral valve, increasing size of the vegetation under antibiotic therapy, certain microorganisms (*S. aureus*, *S. bovis*, *Candida* spp), previous embolism, and multivalvular IE [4]. After initiation of antibiotic therapy, the risk of new embolic events decreases to 6-21% [4].

Because the central nervous system and splenic infarctions are the most common, abdominal and cerebral CT scanning is helpful in detecting septic embolization [4]. Additionally, if applied systematically, CT can detect asymptomatic embolic lesions in 20% of cases [9]. Symptomatic neurological events occur in 15-30% of IE cases, stroke being the most common neurological complication [4]. Additionally, it is associated with increased morbidity and mortality [4]. Splenic infarcts are common and generally asymptomatic [4].

The risk of new embolism is highest during the first days after initiation of antibiotic therapy and decreases after two weeks [4]. The role of surgery in preventing embolization is controversial, but it is more beneficial during the first two weeks of antibiotic therapy [4]. The decision to perform surgery should take into account the operative risk, clinical status, and comorbidities of the patient and is indicated for vegetations size greater than 10mm after embolic events, despite appropriate treatment [4].

Coronary artery septic embolization occurs in 0.31-0.51% of patients with IE [5]. The most common cause of acute coronary syndrome (ACS) in IE is coronary embolism [7]. Other possible causes are occlusion of the coronary ostium by vegetation, decreased coronary artery perfusion in the setting of aortic insufficiency, and the preexistence of coronary arteriosclerotic lesions that become apparent during infection [7,10]. Disturbances of hemostasis and activation of vascular platelet elements can also contribute to ACS [10]. Risk factors for coronary embolism are the existence of mobile vegetations, vegetation size greater than 10mm, *Staphylococcus aureus *and streptococcal infections, and previous embolisms [10,11]. 

ACS usually occurs in the first 15 days of disease and the presentation and clinical course are similar to cases associated with arteriosclerotic coronary arteriopathy [7,11]. It is common for patients to have embolisms at other sites and most cases of coronary embolization occur in the LAD artery [7,10].

There are no guidelines for the management of ACS due to IE [3]. Treatment options for coronary artery septic embolization include medical therapy (anticoagulation and fibrinolysis), percutaneous coronary angioplasty, balloon or surgical embolectomy, coronary artery bypass graft (CABG), and transcatheter septic embolus aspiration [5,6,11]. Medical treatment is probably not effective, because of the small proportion of fibrin and platelets in septic emboli, and can be complicated by hemorrhagic events [6,7,11]. Percutaneous coronary angioplasty and balloon embolectomy can result in emboli dislodgment and aneurysm formation, with subsequent coronary rupture [11]. 

Surgical options include debridement, valve replacement, and coronary artery bypass [6]. However, surgical embolectomy can result in bacterial myocarditis and ventricular rupture [11]. Current evidence suggests that CABG may be the best option for reperfusion of the myocardium in patients with IE and should be associated with valve replacement [11]. 

Our patient presented with fever, constitutional symptoms, and signs of neurological involvement, which was consistent with central nervous system embolization. There was no pathogen identified. However, the fact that the patient was stung by a sea urchin and had an infected lesion on the hand made a bacterial cutaneous pathogen the most probable etiological agent and justified the antibiotic therapy coverage. Azithromycin can be included in the empirical treatment, in order to cover *Bartonella* species, which are the second most common cause of culture-negative IE [12,13].

He presented ACS 15 days after disease onset, which was consistent with the fact that most new embolisms, including coronary, occur in the first two weeks of disease. Additionally, the fact that 20-50% of septic embolization may be silent supports the asymptomatic ACS [4]. Therefore, it is recommended to perform coronary angiography in patients with active IE who present elevated troponins or acute left ventricular dysfunction [11]. The indications and timing for surgery remain controversial, but the coexistence of ACS and IE should encourage a combined procedure, such as CABG, in order to address both situations [11]. 

## Conclusions

Infective endocarditis is an infrequent multisystem disease that can be associated with life-threatening complications, such as septic embolization. The fact that coronary artery embolization is very rare and may be silent makes its diagnosis and treatment challenging. Patients with infective endocarditis should be carefully monitored, especially in the first two weeks of disease. Clinical or laboratorial evidence of cardiac distress or changes in the electrocardiogram should prompt the realization of a transthoracic echocardiography and, if appropriate, coronary angiography. Although there are no recommendations regarding the most adequate treatment for patients with infective endocarditis who develop acute coronary syndrome, it has been encouraged to proceed to surgical coronary revascularization and valve replacement, considering this the most effective procedure in addressing both situations.
